# Disclosing Pathogenic Variant Effects on the Structural Dynamics of the VAPB MSP Domain Causing Familial ALS

**DOI:** 10.3390/ijms26136489

**Published:** 2025-07-05

**Authors:** Md Abul Bashar, Nayan Dash, Sarmistha Mitra, Raju Dash

**Affiliations:** 1 Department of Pharmacy, Faculty of Biological Sciences, Islamic University, Kushtia 7003, Bangladesh; bashar.iu.ph@gmail.com; 2 Department of Integrative Biotechnology, College of Biotechnology and Bioengineering, Sungkyunkwan University, Suwon 16419, Republic of Korea; nayandash.1998@gmail.com; 3 Department of New Biology, Daegu Gyeongbuk Institute of Science and Technology, Daegu 42988, Republic of Korea; sarmisthacu@gmail.com

**Keywords:** vesicle-associated membrane protein (VAMP)-associated protein B (VAPB), major sperm (MSP) domain, amyotrophic lateral sclerosis 8 (ALS8), molecular dynamics (MD) simulation

## Abstract

Vesicle-associated membrane protein (VAMP)-associated protein B (VAPB) serves as a tethering factor that interacts with various proteins and recruits these proteins to the ER surface, exerting multiple functions, such as organelle membrane tethering, lipid transfer between organelles, regulation of calcium homeostasis, autophagy, and the unfolded protein response (UPR). Its interaction is often mediated by its MSP (major sperm) domain, which binds with FFAT (two phenylalanines in an acidic tract)-motif-containing proteins. However, pathogenic variations, such as P56S, P56H, and T46I, in the VAPB MSP domain lead to the familial form of amyotrophic lateral sclerosis (ALS8). Still, the underlying pathophysiology of ALS8 due to pathogenic variations in the VAPB MSP domain remains elusive. In this study, we conducted molecular dynamics (MD) simulations to understand the pathogenic-variant-derived changes in the structural dynamics of the VAPB MSP domain. We found that pathogenic variants altered the fluctuations and conformational dynamics of the VAPB protein. Analyzing the organizations of the secondary structure revealed that pathogenic variants changed the composition of secondary structure elements, especially increasing the proportion of α-helix while reducing β-sheet formation, which might affect the organelle tethering and other functions of VAPB, as well as VAPB homodimer and heterodimer formation. Taken together, these findings can be further investigated through in vivo and/or in vitro studies to not only clarify the pathophysiology of ALS8 resulting from VAPB MSP domain pathogenic variants but also develop novel therapeutics for the disease that restore the native structural organizations as well as fluctuations and motions.

## 1. Introduction

**Figure 1:**
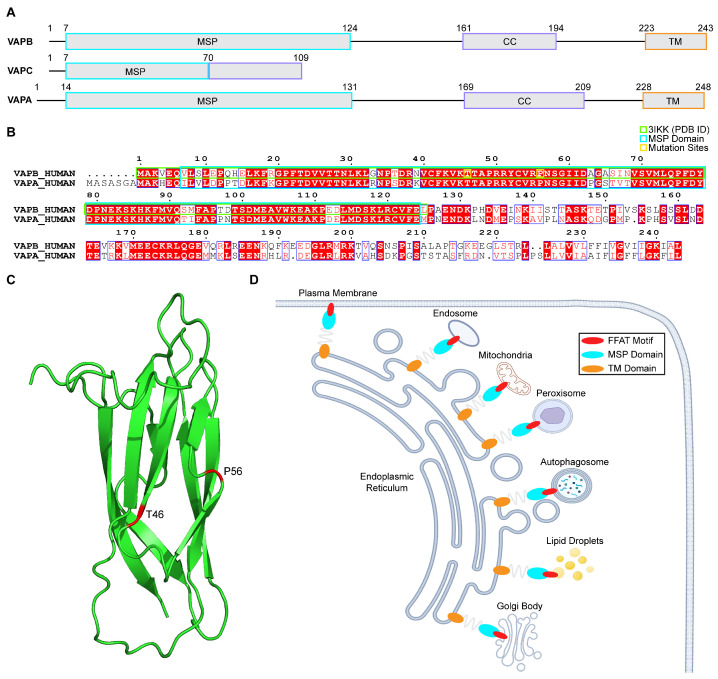
Domain organization of vesicle-associated membrane protein (VAMP)-associated protein B (VAPB), C (VAPC), and A (VAPA); the sequence mapping of two VAPs, i.e., VAPB and VAPA, found in the human genome; the structure of human VAPB major sperm (MSP) domain; and protein–protein interactions between VAPB MSP domain and FFAT-motif containing proteins. (**A**) Visual representations displaying the domain organizations of VAPA, VAPB, and VAPC. Both VAPA and VAPB consist of an N-terminal MSP domain, a coiled-coil (CC) domain, and a C-terminal transmembrane (TM) domain, while VAPC contains only the MSP domain and lacks the CC and TM domains. The MSP, CC, and TM domains are demonstrated as grey boxes outlined in light blue, purple, and orange, respectively. (**B**) The pairwise sequence alignment of two VAPs (i.e., VAPA and VAPB) found in the human genome. The MSP domain sequence is highly conserved between VAPA and VAPB. The sequence mapping of the PDB structure (PDB ID: 3IKK) used in this study is indicated using a green bracket and ranges from sequence 1 to 125, whereas the sequence of the MSP domain is indicated using a turquoise bracket and ranges from sequence 7 to 124. (**C**) The structure of the VAPB MSP domain (PDB ID: 3IKK) is displayed, highlighting the pathogenic variant sites, i.e., T46 and P56. (**D**) Visual representations showing the protein–protein interactions between the VAPB MSP domain and FFAT-motif containing proteins, such as the plasma membrane, mitochondria, the Golgi body, the endosome, the peroxisome, the autophagosome, and lipid droplets.

Vesicle-associated membrane protein (VAMP)-associated proteins (VAPs) are tail-anchored proteins of the endoplasmic reticulum, which function as a tethering factor that is reported to form contact sites between the endoplasmic reticulum (ER) and other organelles, e.g., the golgi apparatus, mitochondria, endosomes, peroxisomes, transport vesicles, lipid droplets, and autophagosomes, at membrane contact sites (MCSs) to maintain their structure and function (Figure 1D) [1,2]. The human genome possesses two VAPs (VAPA and VAPB), which consist of an immunoglobulin-like fold comprising a seven-stranded β sandwich and one α helix, also known as the N-terminal major sperm (MSP) domain, which is followed by a coiled-coil domain (CC) and a C-terminal transmembrane domain (TM), a hydrophobic sequence that anchors the VAPs to the ER bilayer (Figure 1A–C) [3,4,5]. VAPA and VAPB share 63% sequence identity, especially in the MSP domain (82% identity) (Figure 1B) [3,6]. Besides forming homodimers (VAPA-A and VAPB-B dimers), two VAP proteins can form heterodimers (VAPA-B dimers) because of the interactions of their TM domains [3,7]. The *VAPB* gene is located at locus 20q13.3 and encodes a protein consisting of 243 amino acids (Figure 1A,B) [3,8]. In mammals, besides VAPA and VAPB, an alternatively spliced isoform of VAPB was also discovered and termed VAPC. It consists of the first 70 amino acids of the MSP domain of VAPB and an additional unrelated 29 amino acids, lacking both the CC and TM domains of VAPB (Figure 1A) [3,6]. In addition, five different VAPB splice variants were detected at the mRNA level, although translation products of these transcripts were undetectable in tissues [3,6].

As already stated, VAPB is reported to interact with many proteins and recruit them to the ER surface (Figure 1D), exerting various functions, such as organelle membrane tethering [1], lipid transfer between organelles [9,10], regulation of calcium homeostasis [11,12], autophagy [13], and the unfolded protein response (UPR) [7,14]. These interactions and recruitments are often mediated by the binding of the VAPB MSP domain to the FFAT (two phenylalanines in an acidic tract—consensus sequence: EFFDAXE, where X denotes any amino acid motif in the ligand (Figure 1D)—where FFAT motifs bind transversely to the electropositive surface across four of the seven β strands, flanked by two hydrophobic pockets [5,15,16,17]. However, not all VAPB binding partners contain the FFAT motif, in which case they interact with regions of the VAPB MSP domain distinct from the FFAT binding site [18].

VAPB has been reported to be associated with multiple neurodegenerative diseases, such as amyotrophic lateral sclerosis (ALS), Alzheimer’s disease (AD), α-synucleinopathy, Parkinson’s disease (PD), and multiple system atrophy (MSA), which results from either mutation in VAPB, reduction in its levels, or disruption of its interaction with PTPIP51 [18]. The missense variant p.Pro56Ser (also denoted as P56S) in VAPB has been reported to be associated with ALS8, which was first found in Brazilian families of Portuguese descent [19,20] and later identified in German, Japanese, Chinese, and North American families [21,22,23,24]. The presence of the pathogenic variant has been associated with a phenotype characterized by lower motor neuron involvement, with symptoms including progressive muscle weakness (mainly in the lower limbs), muscle atrophy, cramps, tremors, fasciculations, pain, abdominal protrusion, autonomic dysfunction (e.g., choking, constipation), and subtle cognitive and behavioral impairments [19,20,21,23,24,25,26,27,28,29,30,31,32,33]. It has been reported that P56S makes the MSP domain unstable, not only making VAPB^P56S^ aggregation-prone but recruiting coexpressed VAPB^Wild^ and VAPA to the aggregation, negatively affecting VAP’s normal functions [7,34,35,36,37]. This may be due to the substitution of the Pro56 residue, which is located in the MSP domain, which, in turn, as mentioned above, is crucial for making contacts with FFAT-containing ligands and for the correct folding of the seven β-strands of the MSP domain [17]. VAPB^P56S^ is also associated with inducing ER stress [38,39] and disrupting cellular homeostasis [25,40] and autophagy [13,41]. Another missense variant, p.Pro56His (also expressed as P56H), is associated with clinical features similar to those of P56S patients [28]. An additional pathogenic variant in the VAPB MSP domain is p.Thr46Ile (also defined as T46I), which has also been associated with familial ALS [42]. Two other missense variants reported, located outside the MSP domain, were p.Ala145Val [43] and p.Val234Ile [44]. Of these, variants in the MSP domain have been widely studied, as the MSP domain is directly associated with forming contacts with proteins. Although VAPA and VAPC share a conserved amino acid sequence with VAPB, including a sequence from the MSP domain, there is no clinical data that shows the pathogenic variation effects of VAPA and VAPC. Therefore, until now, many studies have tried to disclose how the pathogenic variants in the MSP domain of VAPB trigger ALS8. In this regard, Kumar et al. [45] carried out a 25 ns long molecular (MD) simulation study and reported that SNPs, including P56S and T46I, reduce the protein stability and cause its aggregation. However, this study could not disclose the atomic mechanism of how these pathogenic variants reduce the protein’s stability and cause aggregation. Hence, a comprehensive MD simulation study is indispensable to resolve the atomic mechanism of how these pathogenic variants in the MSP domain reduce the protein’s stability and trigger aggregation. This study employed a 2.5 μsMD simulation followed by comprehensive analyses to elucidate the alteration in structural dynamics and secondary structure organization of VAPB due to the pathogenic variants and how those alterations are associated with the pathophysiology of ALS8.

## 2. Results

In this study, we employed a 2.5 μs timescale MD simulation to elucidate how pathogenic variants (T46I, P56H, and P56S) in VAPB MSP domains alter the structural dynamics of the VAPB MSP domain by comparing with the wild VAPB MSP domain. For each condition (i.e., wild, T46I, P56H, and P56S), five independent MD runs, each lasting 500 ns (2.5 μs altogether), were executed, and Figure S1 displays the extracted root-mean square deviation (RMSD) plots for each independent run. The RMSD plots in Figure S1 confirmed that wild and pathogenic variants retained equilibration throughout the simulation period in all replicates, signifying their adequacy for conformational sampling. Additionally, utilizing cosine content analysis for the first five principal components, we further verified the suitability of conformational sampling of each subtrajectory. This demonstrated that cosine content values were notably lower than 0.7 (Table S1), suggesting the convergence of conformational sampling in all subtrajectories. Therefore, these trajectories were deemed suitable for further analysis.

### 2.1. Pathogenic Variants Induce Conformational Alterations in the VAPB MSP Domain

**Figure 2:**
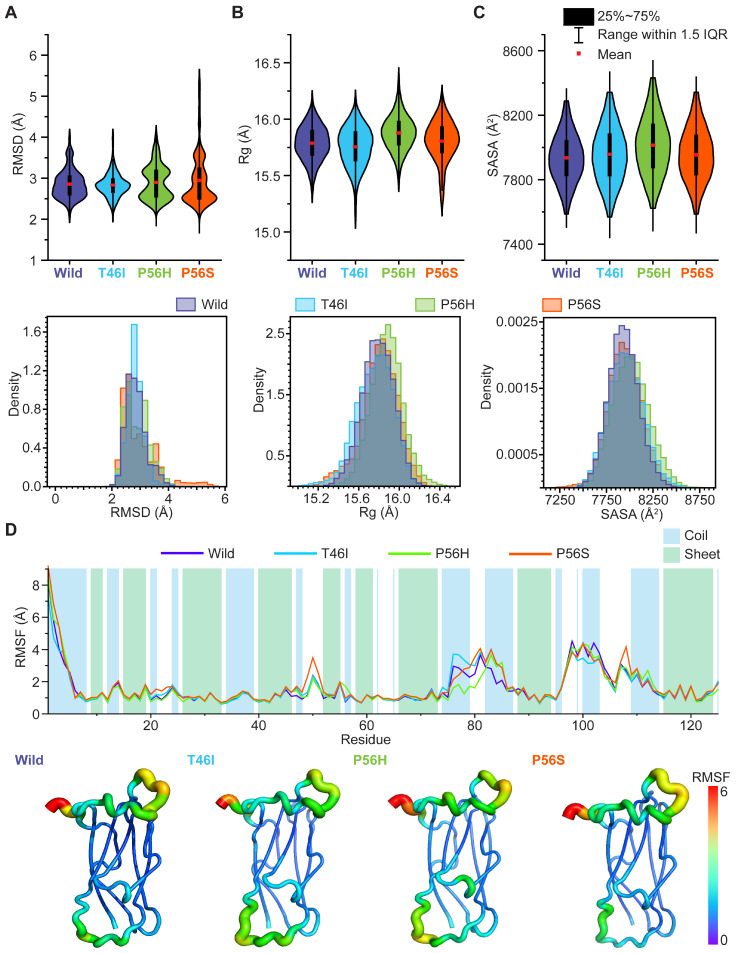
Pathogenic variants alter the conformational dynamics of the VAPB MSP domain. The graphical representations depict the differential conformational changes in the VAPB MSP domain between wild and pathogenic variants, as visualized by (**A**) RMSD, (**B**) Rg, and (**C**) SASA using violin (upper panel) and distribution plots (bottom panel). The residual alterations in the VAPB MSP domain are illustrated by employing (**D**) a line plot (upper panel) and a rainbow color-coded tube depiction (bottom panel), which are based on Cα-root mean square fluctuation (RMSF). The blue and green colors symbolize the coiled-coil and β-sheet of the VAPB MSP domain, respectively (**D**; upper panel). Regions with low RMSF are portrayed by a violet-highlighted narrow tube; in contrast, regions with high RMSF are illustrated by a red-highlighted large tube (**D**; bottom panel).

Before proceeding with the analysis, dictating how pathogenic variants render the VAPB MSP domain unstable, it was crucial to understand if the pathogenic variants induced conformational alterations of the VAPB MSP domain. When we analyzed the effects of pathogenic variants, we observed that P56H induced conformational flexibility in the MSP domain (Figure 2A), which resulted in enhanced spatial distribution of the domain’s atoms (Figure 2B) and an increase in solvent-accessible surface area (SASA) (Figure 2C), indicating exposure of hydrophobic amino acids close to water molecules by P56H. Meanwhile, a minor increase in domain flexibility was observed for P56S (Figure 2A), which led to a slight increase in the domain’s compactness and SASA (Figure 2B,C). T46I, on the other hand, despite showing a slight reduction in the domain’s flexibility (Figure 2A) and compactness (Figure 2B), displayed a nominal increase in SASA (Figure 2C), indicating that residues were closely packed in the interior of the domain.

When we analyzed the root-mean square fluctuation (RMSF), which depicts the individual residues’ fluctuations, we observed that all pathogenic variants altered the residual fluctuations of several amino acid residues located in the coiled-coil and β-sheets of the MSP domain. All variants induced fluctuations in Thr46, Thr47-Ala48, and Lys83-Lys85 (Figure 2D). P56H stabilized Phe76-Asp79, while T46I increased the fluctuations of these residues (Figure 2D). On the other hand, P56S stabilized the first two residues, Phe76 and Asp77, while inducing fluctuations in the latter two, Tyr78 and Asp79 (Figure 2D). Furthermore, T46I stabilized the four residues Ser100-Glu103, whereas P56H and P56S induced fluctuations in Asp101 but stabilized the residues Met102-Glu103 (Figure 2D). In addition, P56S induced fluctuations in Gly20-Pro21 and Pro56, whereas P56H increased the fluctuations of Pro111-Glu112 (Figure 2D). These results collectively indicate that pathogenic variants (T46I, P56H, and P56S) induce conformational alterations in the VAPB MSP domain.

### 2.2. Pathogenic Variants Alter the Dynamic Correlated and Anticorrelated Motions of the VAPB MSP Domain

**Figure 3:**
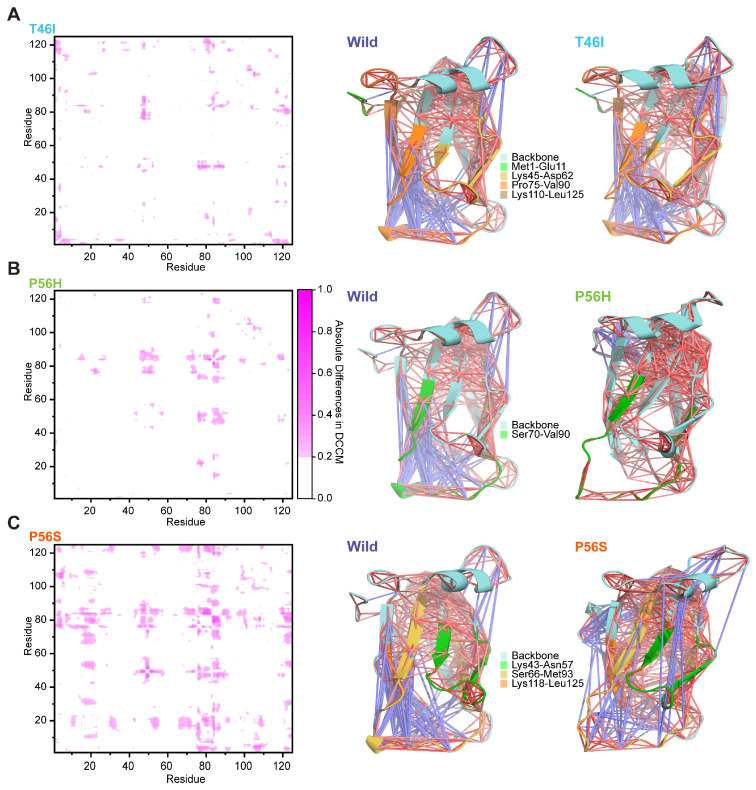
Pathogenic variants alter the correlated and anticorrelated motions of the VAPB MSP domain. The graphical representations (in the left panel) depict absolute differences in the dynamic cross-correlation matrices (DCCMs) between wild and pathogenic variants (T46I, P56H, and P56S). Absolute DCCM differences of equal to or more than 0.2 are portrayed in pink. Plot (**A**) in the left panel indicates the absolute difference in the DCCM matrices between wild and T46I, while plots (**B**) and (**C**) (left panel) denote the absolute differences in the DCCM matrices between wild and P56H and wild and P56S, respectively. The representations (right panel) display structural snapshots of the DCCM between wild and T46I (**A**), wild and P56H (**B**), and wild and P56S (**C**), with blue lines (−1) denoting negatively correlated motion and red lines (+1) denoting positively correlated motion. The intensity of the colors reflects the magnitude of strong correlation or anticorrelation. The backbone of the VAPB MSP domain is displayed in cyan, whereas the regions with DCCM alterations are illustrated in green, yellow, and orange.

Since we observed that pathogenic variants alter the conformational changes in the VAPB MSP domain, we assumed they may also alter the dynamic motions of the VAPB MSP domain. Hence, we considered whether the pathogenic variants affect the inter- and intraresidual correlated motions of the VAPB MSP domain. We noticed that pathogenic variants notably altered the correlated and anticorrelated motions in the VAPB MSP domain and that these alterations were distinct for each pathogenic variant (Figure S2). T46I and P56S induced correlated and anticorrelated motions among the residues in the VAPB MSP domain, whereas P56H abolished these correlated and anticorrelated motions to random motions (Figure S2). We additionally considered the absolute differences in dynamic cross-correlation matrices (DCCMs) between the wild and each pathogenic variant to better understand the effect of pathogenic variants and, more importantly, to detect the amino acid regions that experienced notable shifts in correlated and anticorrelated motions by pathogenic variants and plotted the absolute difference matrix in Figure 3 (left panels), which illustrated pathogenic-variant-induced alterations in correlated and anticorrelated motions of several regions in the VAPB MSP domain, including residues from coiled-coil and β-sheet regions. T46I altered the motions of four regions in the VAPB MSP domain, such as 1–11, 45–62, 75–90, and 110–125 (Figure 3A), whereas P56H changed the motions of the 70–90 region (Figure 3B). P56S, however, caused a more pronounced change in motions than other pathogenic variants; it changed the motion of three regions of the VAPB MSP domain, 43–57, 66–93, and 118–125 (Figure 3C). Collectively, these results indicate that pathogenic variants alter the native correlated and anticorrelated motion of the VAPB MSP domain and that P56S causes the most notable alterations.

### 2.3. Pathogenic Variants Alter the Residual Conformational Motions

**Figure 4:**
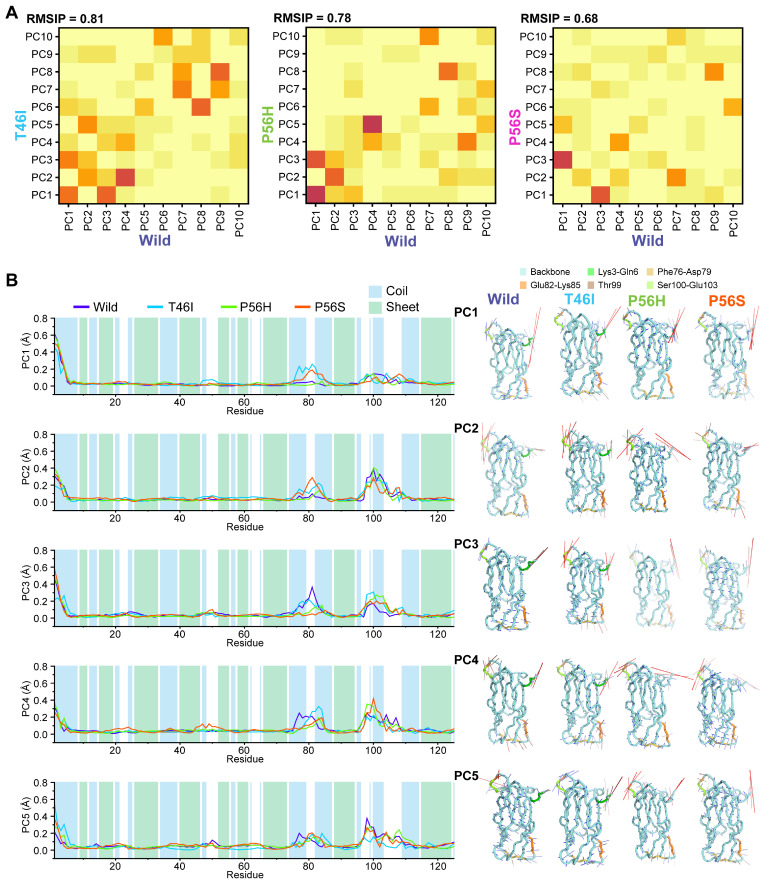
Pathogenic variants alter the residual conformational motions. (**A**) The root-mean-square inner product (RMSIP) data for the first 10 PCs were computed and are illustrated as a gradient heat map to show the similarities and distinctions in the conformational spaces of the wild and each pathogenic variant, with yellow representing a low value and dark red representing a high value. (**A**) The gradient heatmaps of T46I, P56H, and P56S were compared with wild and are displayed in the left, middle, and right panels, respectively. (**B**) The left panel displays line plots illustrating the degree of mobility captured by PC1, PC2, PC3, PC4, and PC5 of the wild and pathogenic variants (T46I, P56H, and P56S). The coiled-coil and β-sheets are depicted using blue and green, respectively. (**B**) The differential contributions of PC1 to PC5 between the wild and pathogenic variants (T46I, P56H, and P56S) are displayed using porcupine plots in the right panel. The length and direction of mod vectors depict the magnitude and direction of structural movements in the VAPB MSP domain, with the backbone structure displayed in cyan and colors ranging from green to yellow to orange highlighting the changes in motion of various regions in the VAPB MSP domain.

Since the pathogenic variants alter the dynamic correlated and anticorrelated motions in the VAPB MSP domain, we sought to conduct principal component analysis (PCA) to understand the dominant motions that varied between wild and pathogenic variants and to disclose the altered collective motions of residual fluctuations with the help of PCs. Consistent with the DCCM results, a pairwise comparison of the best 10 PCs between wild and pathogenic variants harnessing RMSIP calculations confirmed that the dynamic motions differed between wild and pathogenic variants (Figure 4A). The majority of the protein conformational shifts were recorded by PC1, accompanied by PC2 and PC3 (Figure S3). Interestingly, PC1 of P56S captured a higher proportion of the VAPB MSP domain dynamics than PC1 of wild, while T46I and P56H had PC1 values close to wild (Figure S3). When we analyzed the first five principal components using line plots, we found that all pathogenic variants displayed notable fluctuations in PC1 to PC5, specifically in several amino acids from the coiled-coil and β-sheet regions of the VAPB MSP domain (Figure 4B; left panel). All pathogenic variants showed fluctuations in several common regions, including the 76–79, 82–85, 99, 100–103 regions, while T46I additionally demonstrated another fluctuation in the 3–6 region in the VAPB MSP domain (Figure 4B; left panel). Since all PCs captured substantial fluctuations in several regions, we analyzed them using porcupine plots to disclose how they altered the direction of structural shifts in these regions. As shown in Figure 4B (right panel), pathogenic variants altered the magnitude and/or direction of structural shifts across the regions found in the line plots of the PCs. Altogether, these results suggest that pathogenic variants may change the conformational motions of several regions in the VAPB MSP domain.

### 2.4. Pathogenic Variants Alter the Organization of Secondary Structure Formation in the VAPB MSP Domain

**Figure 5:**
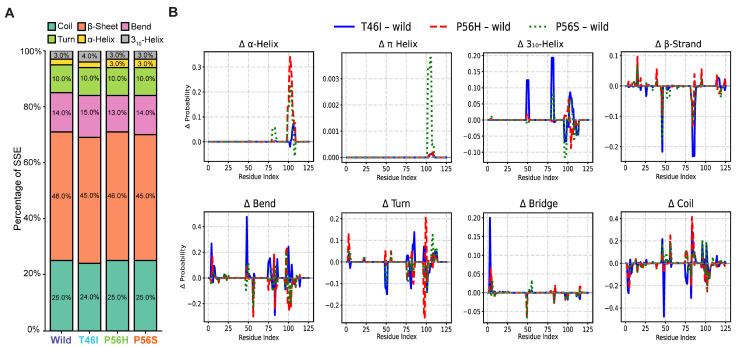
Pathogenic variants alter the organization of secondary structure in the VAPB MSP domain. (**A**) A stacked bar plot generated via the DSSP algorithm demonstrating the percentages of different secondary structural elements (SSE) occupancies, including α-helix, π-helix, bend, β-sheet, β-bridge, coil, and turn. (**B**) The graphical representations show the differences between wild and pathogenic variants (T46I, P56H, and P56S) in the proportion of alterations in secondary structural elements of the VAPB MSP domain.

As we observed that pathogenic variants altered the conformational dynamics and conformational motions, we next sought to elucidate if they changed the formation of secondary structures during simulation. Utilizing the DSSP algorithm revealed that they had differential effects on secondary structure formation (Figure 5A), including alterations in α-helix and β-sheet formation. As shown in Figure 5A, each pathogenic variant induced the formation of either α- or π-helices. For instance, T46I increased π-helix formation by 1%, whereas P56H and P56S increased α-helix content (Figure 5A). In contrast, T46I and P56S reduced β-sheet formation by 1%, while P56H did not alter it (Figure 5A). Besides these alterations, T46I reduced coil formation by 1% but increased bend regions by 1%, whereas P56H reduced bend regions by 1% (Figure 5A). In order to gain further insights and disclose the exact amino acid location where the pathogenic variant altered the secondary structure formation, we created a line plot that shows the specific amino acid position where changes in secondary structure occurred (Figure 5B). T46I showed an increase in α- and 3_10_-helices in residues 48–51, 78–81, and 101–108, whereas P56H showed an increase in α-helices in residues 99–110 but a decrease in 3_10_-helices in residues 105–111 (Figure 5B). P56S, unlike the other pathogenic variants, showed an increase in π-helices in residues 101–109 and a decrease in 3_10_-helices in residues 96–99 (Figure 5B). We also assessed the effect of the pathogenic variation in the β-sheets and β-strands and found that P56S did not notably reduce the proportion of β-sheets, while it reduced the proportion of β-strands in residues 15–16 and 44–46 (Figure 5B). The effect of P56H was less regarding the alteration of β-sheets and β-strands, which it reduced in residues 46–47, 49, and 81–84 (Figure 5B). T46I, on the other hand, demonstrated major reductions in residues 2–3, 46–47, and 82–86. Besides this, the pathogenic variants affected bend, turn, and coil regions (Figure 5B). For example, P56H reduced the turn proportion in residues 97–99, whereas T46I reduced the coils in residues 48–49 but increased the bends in residues 48–49 (Figure 5B). Altogether, these results indicate that pathogenic variants alter the proportions of secondary structures; specifically, they reduce the proportion of β-sheets and β-bridges but increase the proportion of helices.

### 2.5. Pathogenic Variants Alter the Conformation of the Energy-Minima Basins of the VAPB MSP Domain

**Figure 6:**
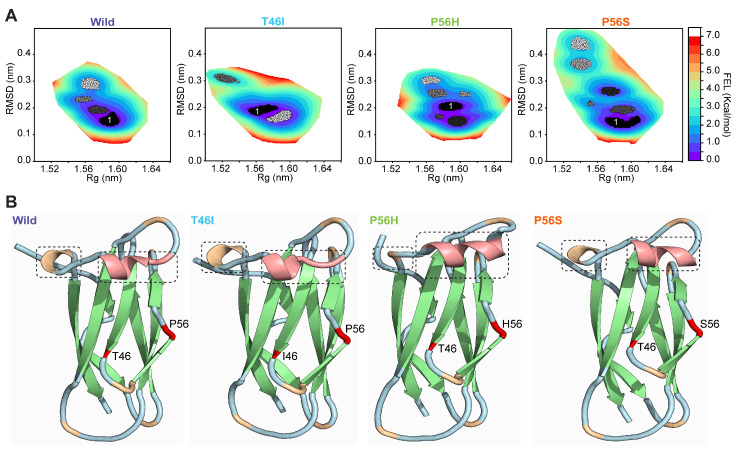
Pathogenic variants change the conformation of the energy-minima basin of the VAPB MSP domain. (**A**) The free energy landscape (FEL) is demonstrated using 2D contour maps for wild, T46I, P56H, and P56S in which the energy-minima basins are represented using black-to-gray gradients, with the darkest color (black) indicating the lowest energy basins (also marked as 1). (**B**) The average structure of each cluster marked as 1 from wild and pathogenic variants (T46I, P56H, and P56S) is represented using 3D cartoon models, where the light blue, green, orange, and red colors in wild and pathogenic variants represent the wild variant’s secondary structures, namely the coil, β-sheets, turn, and α-helix, respectively. The dotted line boxes highlight the secondary structures altered by pathogenic variants. The pathogenic variant sites in each structure are displayed in red.

Next, we tried to gain more insights regarding the effect of pathogenic variants on the conformational dynamics of the VAPB MSP domain utilizing free energy landscape (FEL) analysis for wild and pathogenic variants, where RMSD and Rg were employed as reaction coordinates 1 and 2, respectively. The FEL analysis, which offers an exact precise depictions of the time- and energy-dependent conformation space of proteins by distinguishing between the thermodynamic and kinetic characteristics of wild and pathogenic variants, is represented as a contour map in the Figure 6A, where dark blue regions denote stable minimal-energy states, thus representing energetically favorable protein conformations, while red regions indicate unstable maximal-energy states. We ranked the minimal-energy basins by clustering with respect to FEL value and represented those clusters in Figure 6A, with black to grey gradients, where the black color represents the cluster with the least energy (also marked as 1). As shown in Figure 6A, wild had a total of four energy-minima basins. These were decreased in T46I, which displayed three energy-minima basins. In contrast, P56H and P56S showed six and seven energy-minima basins, respectively (Figure 6A). To better understand how pathogenic variants alter the conformation of VAPB MSP domain, we further visualized the average structure of the clusters with the lowest energies (black color; also marked as 1) for wild and pathogenic variants. As shown in Figure 6B, both P56H and P56S increased the helical content (marked by red color) of the VAPB MSP domain, whereas T46I showed a slight reduction in helical content. In addition, P56H reduced the turns of the VAPB MSP domain, whereas P56S and T46I had negligible effects (Figure 6B). These observations indicate that pathogenic variants, notably P56H and P56S, altered the conformation of the energetically favored state of the VAPB MSP domain.

### 2.6. Pathogenic Variants Alter the Conformational Dynamics of the Energetically Favored State of the VAPB MSP Domain

**Figure 7:**
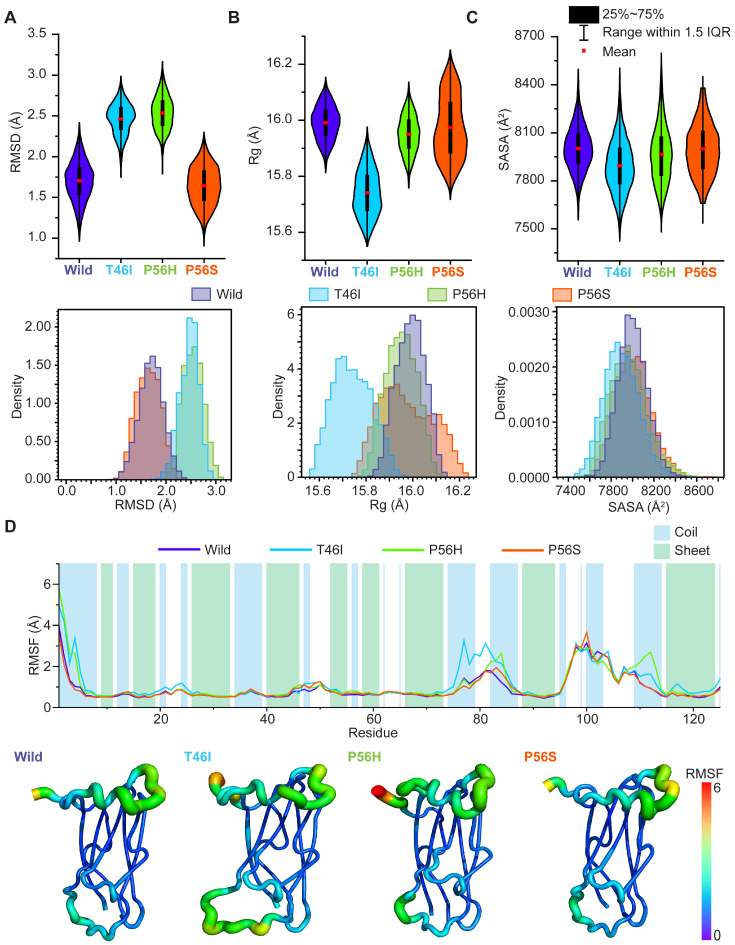
Pathogenic variants change the conformational dynamics of minimal-energy basins of the VAPB MSP domain. Violin (upper panel) and distribution plots (bottom panel) are employed to illustrate the differential conformational changes in the VAPB MSP domain between wild and pathogenic variants in terms of (**A**) RMSD, (**B**) Rg, and (**C**) SASA. Based on Cα-root mean square fluctuation (RMSF), the residual changes in the VAPB MSP domain are shown using (**D**) a line plot (upper panel) and a rainbow color-coded tube representation (bottom panel). The blue and green colors show the VAPB MSP domain’s coiled-coils and β-sheets, respectively (**D**; the upper panel). Narrow tubes with violet highlights represent regions with low RMSF, while large tubes with red highlights represent regions with high RMSF (**D**; the bottom panel).

Since we observed that pathogenic variants might alter the conformation of the minimal-energy basins of the VAPB MSP domain, we next sought to reveal if they changed the conformational dynamics of the VAPB MSP domain and, if so, how and where these changes occurred. When we assessed the effects of T46I and P56H, we discovered that both increased the dynamic nature of the VAPB MSP domain (Figure 7A) but reduced the spatial distribution of the domain’s atoms (Figure 7B), which led to a decrease in SASA value (Figure 7C), suggesting a close packing of hydrophobic amino acids inside the domain. However, the effect of P56H was less intense than that of T46I, especially for Rg and SASA reductions (Figure 7B,C). P56S, on the other hand, showed a slight decrease in RMSD value (Figure 7A), indicating a reduction in the dynamic nature of the domain, which resulted in minor reductions in Rg and SASA values (Figure 7B,C), indicating less spatial distribution of atoms and hence closer packing of residues in the interior of the domain.

Analyzing RMSF results of wild and pathogenic variants disclosed that P56S, consistently with its RMSD, Rg, and SASA results, did not cause notable residual fluctuations in the VAPB MSP domain (Figure 7D). T46I and P56H, however, induced noticeable fluctuations across several residues located in the coil region of the domain (Figure 7D). For instance, both T46I and P56H induced the fluctuations of the residues Met1-Val4, Lys110-Asp113, and Glu82-Lys85, whereas T46I, besides inducing these residues’ fluctuations, increased the fluctuations of Phe76-Asp79 (Figure 7D). These results altogether indicate that pathogenic variants, especially T46I and P56H, may alter the conformational fluctuations in minimal energy basins of the VAPB MSP domain.

### 2.7. Pathogenic Variants Alter the Organization of Secondary Structure in Energy-Minima Basins of the VAPB MSP Domain

**Figure 8:**
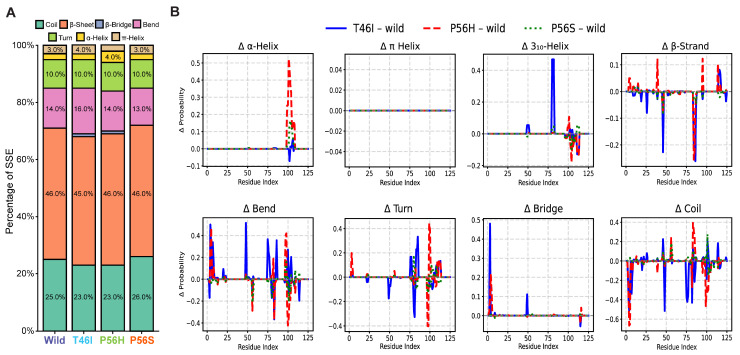
Pathogenic variants change the arrangement of secondary structures in the VAPB MSP domain. (**A**) The DSSP algorithm was employed, and the results are shown as a stacked bar plot indicating the percentages of several secondary structural elements’ (SSEs’) occupancy, where different secondary structural elements, the α-helix, π-helix, bend, β-sheet, β-bridge, coil, and turn, are shown in different colors. (**B**) Graphical representations reveal the variations in the percentage of changes in secondary structural components of the VAPB MSP domain between wild and pathogenic variants (T46I, P56H, and P56S).

Since we observed that all pathogenic variants altered the conformation and conformational dynamics of the VAPB MSP domain, we tried to understand if this alteration was associated with changes in secondary structure organization. As shown in Figure 8A, T46I and P56H reduced the proportion of coils by 2% and demonstrated the formation of β-bridges (Figure 8A), mainly in residues 2–4 (Figure 8B), whereas P56S induced a 1% increase in the proportion of coils (Figure 8A). While P56H and P56S did not change the proportion of β-sheets (Figure 8A), T46I reduced it by 1% (Figure 8A), mainly in residues 47–48 and 84–86 (Figure 8B). T46I increased the formation of bends by 2% (Figure 8A), primarily in residues 3–7, 48–49, 75–77, and 85–86 (Figure 8B), whereas P56S reduced bend formation by 1% (Figure 8A), primarily in residues 57–58, 81–82, and 100–101 (Figure 8B). In addition, T46I increased the proportion of π-helices (Figure 8A), whereas P56H induced the formation of α-helices in residues 99–108 (Figure 8B). Although no pathogenic variant altered the proportion of turn formation (Figure 8A), T46I and P56H altered its formation in some residues (Figure 8B). These results suggest that pathogenic variants, specifically T46I and P56H, change the organization of secondary structural elements in the energy-minima basins of the VAPB MSP domain.

## 3. Discussion

This study employed a comprehensive MD simulation study to investigate how pathogenic variants in the VAPB MSP domain altered its conformational dynamics and conformations linked to ALS8. When we analyzed the pathogenic-variant-derived conformational alterations in the VAPB MSP domain, we found that all variants increased the dynamic nature of the domain (Figure 2A–C), and induced fluctuations in several residues (Figure 2D). For example, T46I raised the fluctuation of Thr46, Thr47-Ala48, Lys83-Lys85, and Phe76-Asp79, but stabilized residues Ser100-Glu103 (Figure 2D). When we studied DCCM and PCA for T46I, we also found that T46I altered the correlated and anticorrelated (Figure 3A), as well as conformational, motion (Figure 4B) of several regions, including those amino acids with altered RMSF (Figure 2D). For example, we found that it altered the correlated and anticorrelated motion of the regions 1–11, 45–62, 75–90, and 110–125 (Figure 3A) and changed the conformational motion of the regions 3–6, 76–79, 82–85, 99, and 100–103 (Figure 4B). These fluctuations and altered motions changed the secondary structure organization in the VAPB MSP domain, which we revealed using the DSSP algorithm (Figure 5A). T46I reduced the proportion of β-sheet formation (Figure 5A), mostly in residues 2–3, 46–47, and 82–86, but increased the proportion of helices in residues 48–51, 78–81, and 101–108 (Figure 5A,B). It replaced the coil in residues 48–49 with a bend (Figure 5B). Additionally, we found that it altered the domain stability of minimal-energy basins (Figure 7), and DSSP analysis revealed that it also reduced beta-sheet content and induced helical content (Figure 8A,B). We concluded that the fluctuations and differential motion across several residues, especially 2–3, 46–47, 48–51, 78–81, 82–86, and 101–108, might increase the helical structures and reduce the β-sheets, and this might be associated with the gain of toxic effects in ALS8.

Examining the effect of P56H showed that it increased the fluctuation of Thr46, Thr47-Ala48, Lys83-Lys85, Asp101, and Pro111-Glu112 but stabilized residues Phe76-Asp79 and Met102-Glu103 (Figure 2D). The results of DCCM and PCA for P56H also revealed that P56H changed the correlated and anticorrelated (Figure 3B) and conformational motion (Figure 4B) in multiple regions, including those amino acids with modified RMSF (Figure 2D). For instance, we discovered that it modified the conformational motion of the regions 76–79, 82–85, 99, and 100–103 (Figure 4B) and changed the correlated and anticorrelated motion of the 70–90 region (Figure 3B). These fluctuations and differential motions changed the secondary structure organization in the VAPB MSP domain, as disclosed by the DSSP algorithm (Figure 5A). P56H increased the alpha-helix proportion in residues 99–110 but lowered beta-sheet formation, especially in residues 46–47, 49, and 81–84 (Figure 5A,B). Furthermore, it lowered turn formation in residues 97–99 (Figure 5B). We also discovered that P56H changed the domain stability of minimal-energy basins (Figure 7) and eliminated turns and increased helical content (Figure 6B); this was confirmed by DSSP analysis as well (Figure 8A,B). We believe that these fluctuations and differential motions across several residues, notably 46–47, 49, 81–84, and 99–110, might be associated with the changes in secondary structure organization, specifically increasing the helical structures and reducing the turns and β-sheets, which, in turn, may be linked to its gain of toxic effects in ALS8.

Observing how P56S affected the VAPB MSP domain revealed that it increased the fluctuations in Gly20-Pro21, Thr46, Thr46, Thr47-Ala48, Pro56, Tyr78-Asp79, and Lys83-Lys85 but stabilized residues Phe76 and Asp77 (Figure 2D). During the analysis of DCCM and PCA for P56S, we identified that it changed the conformational motion (Figure 4B) and correlated and anticorrelated motions (Figure 3C) of various regions, including those amino acids with shifted RMSF (Figure 2D). Specifically, we identified that it had the most potent effect on altering correlated and anticorrelated motion when compared with the other pathogenic variants; it had this effect in the regions 43–57, 66–93, and 118–125 (Figure 3C). It also modified the conformational motion of the regions 76–79, 82–85, 99, and 100–103 (Figure 4B). We additionally found that the PC1 value of P56S covered a larger proportion of motion than that of the wild (Figure S3). However, it showed no noticeable changes in residual fluctuations (Figure 4B), which also explains why P56S has the most deleterious effects in ALS8. The DSSP algorithm revealed that these fluctuations and altered motions altered the secondary structure organization in the VAPB MSP domain (Figure 5A,B). P56S showed increased α-helix content in residues 101–109 (Figure 5A,B) but lowered the percentage of beta-sheet formation, mainly in residues 15–16 and 44–46 (Figure 5A,B). We also discovered that P56S changed the domain stability of minimal-energy basins (Figure 7); more notably, it increased helical content (Figure 6B), as DSSP analysis further confirmed (Figure 8). We assume that the changes in the fluctuations and differential motions across many residues, particularly 15–16, 44–46, and 101–109, may be responsible for the changes in secondary structure, including the increase in helical structures and decrease in beta-sheets, and that these changes in secondary structure organization could be associated with its gain of toxicity in ALS8.

Collectively, our results suggest that pathogenic-variant-derived increases in hydrophobic surface area and alterations in fluctuations and motions across several regions in the VAPB MSP domain altered the organization of secondary structural elements, specifically increasing helical content and reducing the proportion of beta-sheets. These findings are consistent with previous studies, where it was found that P56S increased the hydrophobic surface area [46] and reduced the proportion of β-sheets [17,47]. While amyloid aggregates are primarily characterized by β-sheets, α-helices can indeed play a role in amyloid formation by acting as intermediates. To date, multiple reports have demonstrated that during amyloid formation, unstructured proteins can experience a transition in their structures from random coils to β-sheet via an intermediate enriched with helices [48,49,50]. These proteins include amyloid beta (Aβ) [48], islet amyloid polypeptide (IAPP) [49], and α-synuclein [50]. For example, Ghosh et al. reported that the N-terminal portions of α-synuclein, having a tendency to assume a helical structure, may trigger the gradual formation of helix-rich intermediates via intermolecular interactions. Helix–helix interactions further promote fibril formation; once formed, these fibrils trigger the aggregation of proteins into fibrils [50]. The authors demonstrated that the formation of these helix-rich intermediates is usually initiated during the initial stage of the elongation phase of aggregation, which continues until the mid-elongation phase, when all proteins change into β-sheet structures [50]. We hypothesize that VAPB, like α-synuclein, Aβ, and IAPP, might also undergo this intermediate helix-rich state pathway during its aggregation. Since VAPB is directly involved in forming interactions with proteins and recruiting them to the endoplasmic reticulum, we assume that these pathogenic-variant-derived alterations in secondary structural elements might also interrupt or debilitate protein–protein interactions between the VAPB MSP domain and FFAT-motif-containing proteins, which may affect the organelle tethering and other functions of VAPB. Furthermore, these alterations in structural organization and dynamics and the increased tendency to aggregate might affect VAPB homodimer and heterodimer formation. Altogether, we believe that these alterations might be associated with the gain of toxicity or adverse effects in subjects with pathogenic variants in the VAPB MSP domain (T46I, P56H, and P56S), leading to ALS8.

## 4. Materials and Methods

### 4.1. Preparation of the System for MD Simulation

The 3D crystal structure of the VAPB MSP domain (PDB ID: 3IKK) was extracted from the RCSB protein databank, and according to previous articles [51,52], this structure was subsequently prepared utilizing the Schrödinger 2023-2 program (Schrödinger, LLC, New York, NY, USA). After preparing the protein, we harnessed the residue scanning calculation module within Schrödinger 2023-2 to generate the pathogenic variants (T46I, P56H, and P56S) [53,54].

To comprehend pathogenic-variant-derived alterations in conformational dynamics of the VAPB MSP domain, we executed MD simulations for wild and pathogenic variants utilizing the academic edition of the Desmond program, Schrödinger 2023-2 (Schrödinger, LLC, New York, NY, USA) [55,56]. Following earlier published articles [57,58,59], we modeled the wild and pathogenic variants by applying the OPLS4 force and placed them within an orthorhombic box having dimensions of 10 Å on all sides. We succeeded in solvating wild and pathogenic variants by employing the explicit solvation model, which is also known as the Monte Carlo simulated transferable intermolecular potential with three points (TIP3P) water model. With the introduction of counterions (Na^+^/Cl^−^), we neutralized the system and adjusted the system’s salt concentration to 0.15 M to emulate physiological conditions. Utilizing the default Desmond settings, which include a sequence of constrained minimization phases and MD, each system underwent minimization and equilibration following earlier work [59] before we proceeded with MD simulations. In order to conduct MD simulation under thermodynamic settings, we fixed the temperature at 300 K and the pressure at 1 atm, which was accomplished by harnessing a Nosé–Hoover thermostat [60] and an isotropic Martyna–Tobias–Klein barostat [61]. We set 9.0 Å as a cutoff for assessing short-range electrostatic interactions, whereas the particle mesh Ewald approach was harnessed for electrostatic interactions that were long-range. Integrating the equations of motion for both bonded and nonbonded dynamics was accomplished by harnessing a multistep RESPA integrator using a 2.0 fs inner time step [62]. This integration was conducted within a predetermined short-range cutoff. We harnessed 6.0 fs as an outer time step for nonbonded interactions exceeding the cutoff. Following this, we employed the NPT ensemble technique and subsequently executed MD simulations for the wild and three pathogenic variants. For each system, the simulation was performed 5 times with 500 ns for each time, where the coordinates were captured following every 100 ps, as we set the frame rate at 100 ps. Following the MD simulation, we assessed the stability of the wild and pathogenic variants with the help of trajectories obtained from MD simulation utilizing RMSD (root mean square deviation), RMSF (root mean square fluctuation), Rg (radius of gyration), and SASA (solvent-accessible surface area) with Schrödinger 2023-2. The occupancy of secondary structural elements (SSE) was assessed utilizing the Dictionary of Secondary Structure in Proteins (DSSP) algorithm [63].

### 4.2. Analysis of Protein Dynamics

To comprehend how the motions of amino acid residues changed over time during the MD simulation, we employed the Bio3D R package (version: 2.4.5) to generate dynamic cross-correlation maps (DCCMs) [64]. We implemented the following equation to compute the cross-correlation ratio (C_ij_) between the skeleton’s i and j carbon atoms.

$$C_{\mathrm{ij}}=\frac{\langle \Delta r_{i}\cdot \;\Delta r_{j}\rangle }{{\left\{\langle \Delta r_{i}\rangle^{2}\langle \Delta r_{i}\rangle^{2}\right\}}^{1/2}}$$

Here, the average position of the ith and jth residues is symbolized by ∆r_i_ and ∆r_j_, respectively, whereas the mean time of the entire trajectory is represented by the angled brackets “〈〉”. The results of DCCM ranged from −1 to +1, where positive and negative values represented positively and negatively correlated motion, respectively.

Furthermore, principal component analysis (PCA), which was executed with the assistance of an R program called Bio3D, was utilized to further comprehend the flexibility and collective motions of wild and pathogenic variants. Following the removal of any translational or rotational components, we proceeded to investigate the covariance matrix of atomic coordinates as well as the eigenvectors to determine the values of the PCA eigenvectors. This was followed by the diagonalization of the orthogonal coordinate transformation matrix, which resulted in the production of the diagonal matrix of eigenvalues. These columns were used to depict the eigenvectors that correlated with the direction of motion measured about the initial coordinates. The eigenvector was connected to the eigenvalue and reflected the complete mean-square displacements (MSD) of the system. There have been earlier studies that have mathematically covered further information on the complete system [65,66].

### 4.3. Free Energy Landscape (FEL)

A conformational sampling approach was employed to derive the free energy landscape (FEL), which encompassed every potential macromolecular structural conformation. With the help of Gibbs free energy, protein stability was characterized as a function of entropy and enthalpy in FEL analysis. The accompanying equation was employed to compute FEL:

$$G_{i}=-K_{B}\mathrm{TIn}\;\left(N_{i}/N_{\max}\right)$$

In this context, k_B_ denotes Boltzmann’s constant, whereas G_i_ stands for the Gibbs free energy of the state, K_B_. The temperature, established at 300 K, is represented by the symbol T. N_i_ and N_max_ represent the population bin of I and the most occupied population bin, respectively. The unpopulated bin was designated as having minimal provability according to an artificial scale, in which a color-coded model shows the energy of various levels. The conformer with the lowest energy underwent RMSD, RMSF, Rg, SASA, and DSSP analyses utilizing the MDAnalysis (https://github.com/MDAnalysis/, accessed on 2 May 2025) and MDTraj (https://github.com/mdtraj/mdtraj, accessed on 2 May 2025) packages.

## 5. Conclusions

Illustrating the mechanism behind developing ALS8, which is associated with several toxic effects due to pathogenic variants in the VAPB MSP domain, is indispensable to comprehending how increased hydrophobic surface and altered fluctuations and dynamics motions lead to changes in the secondary structure organization. Utilizing a comprehensive MD simulation study, this study uncovered the key residues whose altered fluctuations and motions may be associated with differential organization in the secondary structures and ultimately with the pathophysiology of ALS8. The observations from this study can be further subjected to in vivo and/or in vitro studies to not only elucidate the pathophysiology of ALS8 caused by VAPB MSP domain pathogenic variants but also develop novel therapeutics for the disorder that restore the native fluctuations and motions.

## Data Availability

All raw data included in this article are accessible to competent researchers upon adequate request.

## Supplementary Materials

The following supporting information can be downloaded at: https://www.mdpi.com/article/10.3390/ijms26136489/s1.
