# Supplementary material for: Disclosing Pathogenic Variant Effects on the Structural Dynamics of the VAPB MSP Domain Causing Familial ALS
Source: Int J Mol Sci. 2025 Jul 5;26(13):6489. doi: 10.3390/ijms26136489 (PMC12249741; doi:10.3390/ijms26136489)
Supplement: Supplementary file 1 [file ijms-26-06489-s001.zip › ijms-3665738-supplementary.pdf]

Disclosing pathogenic variant effects on the structural dynamics of the VAPB MSP domain causing familial ALS

Md Abul Bashar[1], Nayan Dash[2], Sarmistha Mitra[3], Raju Dash[3,*]

[1]Department of Pharmacy, Faculty of Biological Sciences, Islamic University, Kushtia 7003, Bangladesh

[2]Department of Integrative Biotechnology, College of Biotechnology and Bioengineering, Sungkyunkwan University, Suwon, Gyeonggi-do 16419, Republic of Korea

[3]Department of New Biology, Daegu Gyeongbuk Institute of Science and Technology, Daegu 42988, Republic of Korea

***Corresponding Author**

Raju Dash

Department of New Biology

Daegu Gyeongbuk Institute of Science and Technology

Daegu 42988, Republic of Korea

Email: dash_raju@dgist.ac.kr

Tel.: +82-53-785-1730

Table S1. Each sub-trajectory was subjected to the cosine content analysis for the first five principal components.

System	PC1	PC2	PC3	PC4	PC5
Wild	0.006354	0.296999	0.020401	0.00076	0.061896
T46I	0.306436	0.185295	0.052144	0.002009	0.087397
P56H	0.018222	0.281496	0.04036	0.039324	0.01527
P56S	0.255973	0.143338	0.061044	0.010392	0.000627

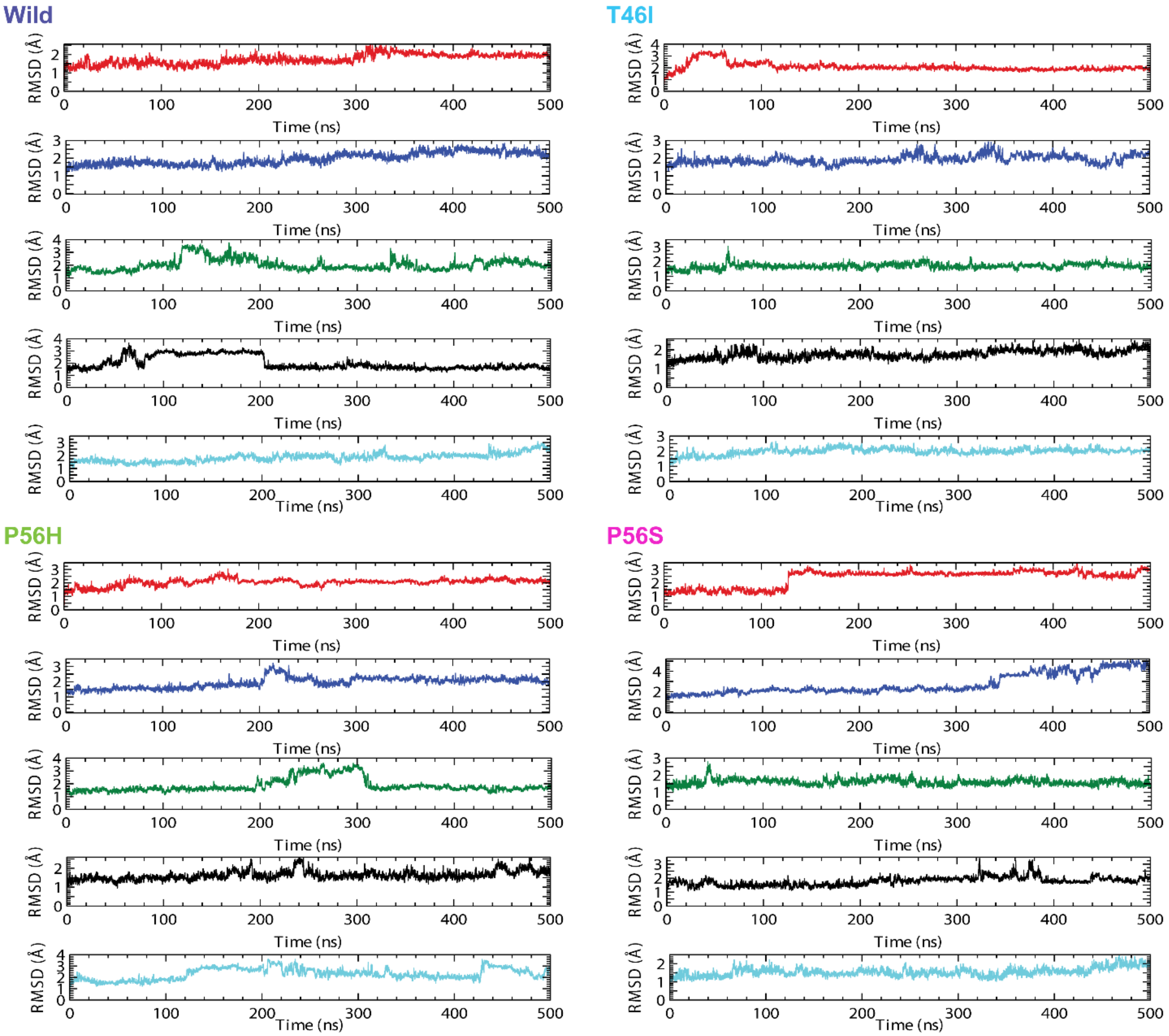

Figure S1. The root mean-square deviation (RMSD) results were assessed for wild and pathogenic variant-containing structures using protein C-alpha of each replicate of Wild, T46I, P56H, and P56S, respectively.

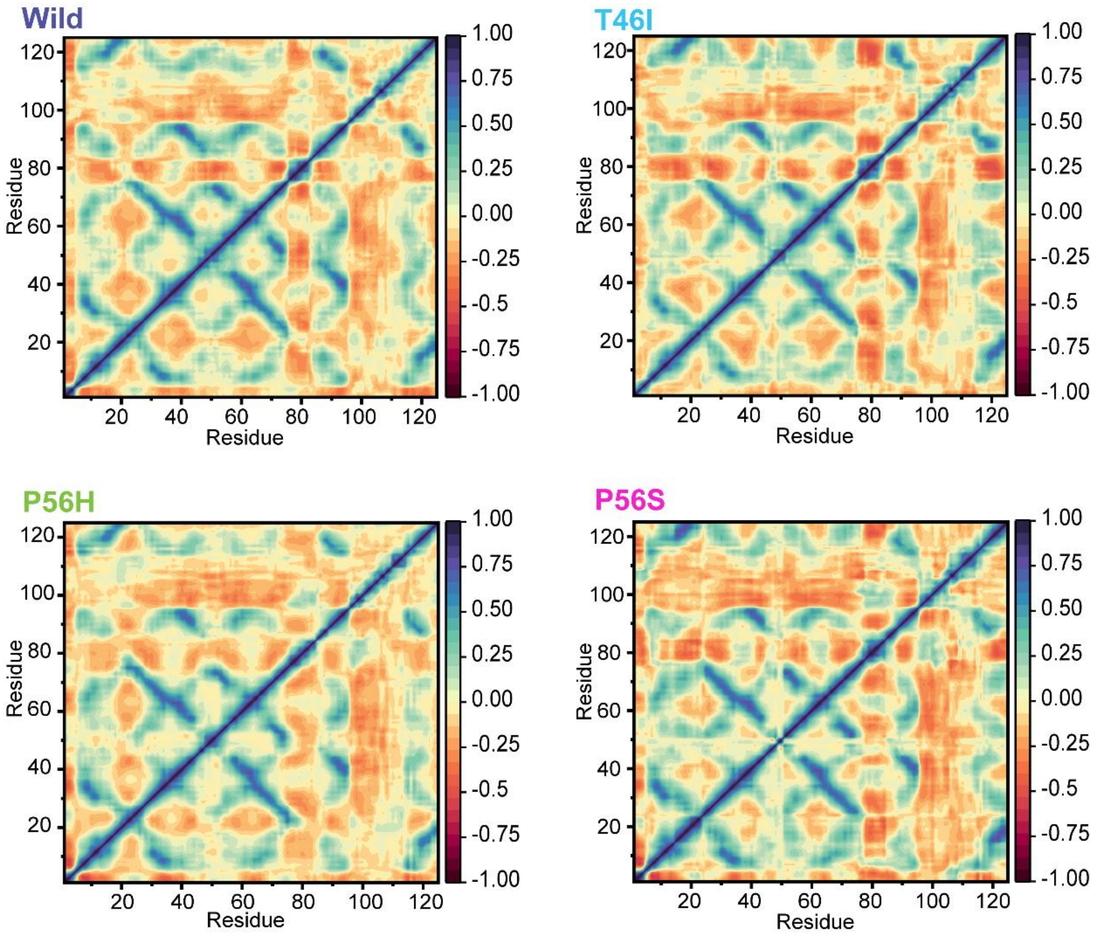

Figure S2. The dynamic cross-correlation matrix, abbreviated as DCCM, is a matrix that represents the anticorrelated and correlated movements between each pair of residues in the structures of wild and three pathogenic variants of the VAPB MSP domain (T46I, P56H, and P56S). The color red (+1) is used to indicate a strongly correlated motion, whereas the blue (-1) represents a motion that is anticorrelated.

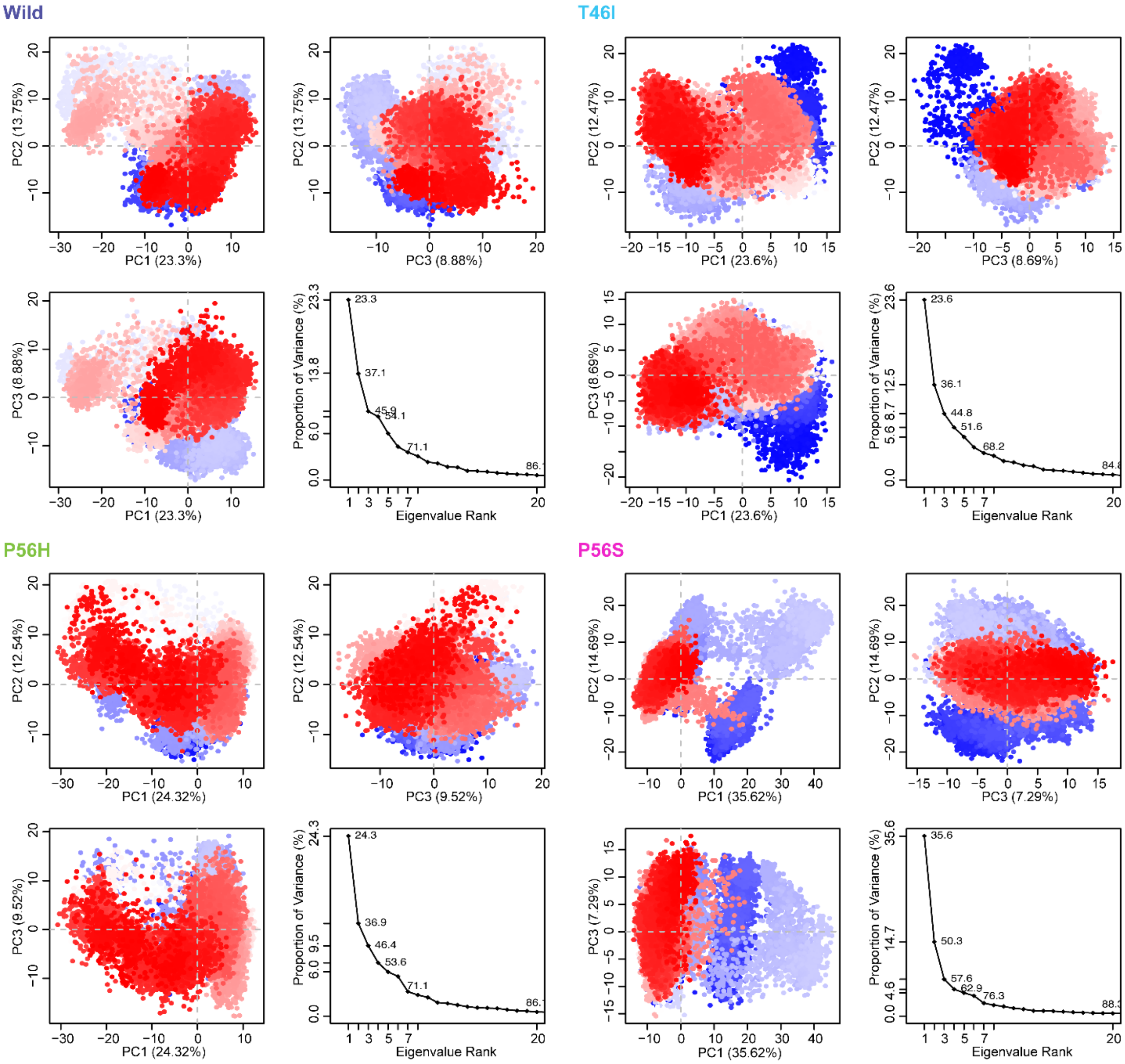

Figure S3. A depiction of the trajectory conformer distribution is displayed across the principal planes components (PC) 1, 2, and 3, with each dot expressing a structure that changed color over the course of time (from blue to red). The first three diagrams illustrate the comparison between PC1, PC2, and PC3 of wild and three pathogenic variants of the VAPB MSP domain (T46I, P56H, and P56S), while the fourth plot illustrates the magnitude of variance for each PC of wild and three pathogenic variants.
